# Sleep Quality as a Modifier of Plasma pTau217 and GFAP Associations with Cognitive Function

**DOI:** 10.1093/geroni/igaf122.4194

**Published:** 2025-12-31

**Authors:** Ramkrishna Kumar Singh, Semere Bekena, Yiqi Zhu, Paris Adkins-Jackson, Beau M Ances, Ganesh Babulal

**Affiliations:** Washington University School of Medicine in St. Louis, St. Louis, Missouri, United States; Washington University School of Medicine in St. Louis, St louis, Missouri, United States; Washington University School of Medicine in St. Louis, Washington Universury, Missouri, United States; Mailman School of Public Health, Columbia University, New York, New York, United States; Washington University School of Medicine in St. Louis, St. Louis, Missouri, United States; Washington University School of Medicine in St. Louis, SAINT LOUIS, Missouri, United States

## Abstract

**Background:**

Plasma biomarkers, such as neurofilament light chain (NfL), glial fibrillary acidic protein (GFAP), phosphorylated tau (pTau217), and total tau (tTau), are associated with cognitive decline. However, the role of sleep quality in modifying these associations remains unclear. This study examines whether subjective sleep quality, as measured by the Pittsburgh Sleep Quality Index (PSQI), modifies the associations between plasma biomarkers and cognitive performance.

**Methods:**

We analyzed cross-sectional data from 491 adults aged 36 years or older in the Aging Adult Brain Connectome study. Plasma levels of NfL, GFAP, pTau217, and total tTau were measured. Cognitive performance was assessed using the Montreal Cognitive Assessment (MoCA) and the Preclinical Alzheimer’s Cognitive Composite (PACC). Sleep quality was measured using the Pittsburgh Sleep Quality Index (PSQI). Generalized linear models were used to test main and interaction effects while adjusting for demographics. Sensitivity analyses included APOE ε4 status and body mass index.

**Results:**

Higher plasma levels of NfL, GFAP, and pT217 were associated with lower cognitive performance on both MoCA and PACC (all P < 0.05). Poorer sleep quality was independently associated with worse PACC outcomes. Critically, significant interaction effects were observed: PSQI moderated the negative associations between GFAP and both PACC (β = 0.0003, P = 0.039) and MoCA (β = 0.0019, P = 0.021), and between pT217 and MoCA (β = 0.0299, P = 0.004), indicating a synergistic relationship between sleep quality and glial/ amyloid-related pathology in cognitive aging.

**Conclusion:**

Sleep quality modifies biomarker-cognition associations, highlighting its potential as a behavioral target to support brain health.

